# Comprehensive Characterization of a Reference Ferroelectric Nematic Liquid Crystal Material

**DOI:** 10.3390/ma18245496

**Published:** 2025-12-06

**Authors:** Ayusha Paul, Milon Paul, Manisha Badu, Arjun Ghimire, Netra Prasad Dhakal, Samuel Sprunt, Antal Jákli, James T. Gleeson

**Affiliations:** 1 Department of Physics, Kent State University, Kent, OH 44242, USA; apaul15@kent.edu (A.P.); mbadu@kent.edu (M.B.); aghimir3@kent.edu (A.G.); ssprunt@kent.edu (S.S.); ajakli@kent.edu (A.J.); 2 Advanced Materials and Liquid Crystal Institute, Kent State University, Kent, OH 44242, USA; milon@kent.edu (M.P.); ndhaka1@kent.edu (N.P.D.)

**Keywords:** liquid crystal, ferroelectric, smectic ZA, polarization, viscosity, dielectric constants, elastic constants, Freedericksz transition

## Abstract

Among the recently developed ferroelectric nematic liquid crystals, FNLC-919, synthesized by Merck Electronics KGaA, stands out for its stable, room-temperature, ferroelectric nematic (N_F_) phase. This renders it a promising candidate for both fundamental research and device-level applications. In this study, we present a comprehensive experimental investigation of FNLC-919, focusing on its structural, optical, dielectric, and elastic properties in the paraelectric nematic (N) and the intermediate antiferroelectric phase (dubbed N_X_) that occur in a temperature range between the N and N_F_ phases. Key material parameters such as ferroelectric polarization, viscosity, and nanostructure are characterized as functions of temperature in all mesophases, while the orientational elastic constants are determined only in the N and N_X_ phases. Our findings are compared with prior results concerning the benchmark compound DIO that also exhibits the phase sequence N-N_X_-N_F_ and reveals a smectic-like mass density wave coinciding with antiferroelectric ordering in the N_X_ phase.

## 1. Introduction

Since the discovery of liquid crystals in 1888 by Friedrich Reinitzer [1], numerous exotic nematic phases such as the chiral, biaxial, bent-core, and twist–bend nematic phase of liquid crystals have been theoretically predicted and experimentally realized, expanding the horizon of soft matter physics [2,3,4]. The traditional nematic phase of liquid crystals is characterized by a long-range orientational order but no positional order or macroscopic polarization. While most compounds that exhibit the nematic phase have non-zero molecular dipole moments, the bulk material remains non-polar because the director ($\hat{n})$ exhibits head-to-tail equivalency [5].

In 2017, two materials were independently reported [6,7] to exhibit the polar nematic phase. Shortly thereafter, these materials were confirmed to possess switchable spontaneous [8,9] polarization, i.e., ferroelectricity [10]; this phase is denoted as the ferroelectric nematic (N_F_) phase. The polar nature of this phase allows for linear electro-optic coupling at fields as low 1 V/mm [10]. The discovery of the long-sought-after N_F_ phase has catalyzed a significant increase in the synthesis [11,12,13,14] and analysis [15,16,17,18,19] of new FNLCs. Furthermore, the N_F_ phase exhibits electrohydrodynamic instability [20,21,22], thermomechanical coupling [23], and filament formation [8,9]. N_F_ materials also show remarkable linear electromechanical responses [24,25,26], large second harmonic generation signals [12,27,28], second-order non-linear optical (NLO) coefficients [29,30], exotic entangled photon-pair generation [31], and bulk photovoltaic effects [32] that hold promise for new technological applications. Many technological applications will become far more accessible with access to stable materials exhibiting the N_F_ phase at ambient temperatures.

In this work, we report comprehensive physical property measurements on one such candidate material, the mixture FNLC-919, prepared and provided by Merck Electronics, KGaA, Darmstadt, Germany. Its phase sequence on cooling was reported as I-80 °C-N-44 °C–N_X_-32 °C-N_F_-8 °C–Cr, where N_X_ is the tentative identifier for the phase that is intermediate between N and N_F_. Although FNLC-919 has been studied by various groups describing their alignment properties and birefringence [33], its ability to form free-standing filaments [8] and its use in electrically tunable lenses and reflectors [34,35,36], a complete characterization of its physical properties and the identification of the nature of its phase between the N and N_F_ phases, are lacking. To fill this gap, we present a detailed experimental investigation of FNLC-919, encompassing complete phase identification, polarization and dielectric constant measurements, orientational elastic constants and viscosity measurements, and results from small-angle X-ray scattering.

## 2. Methods and Results

**Figure 1:**
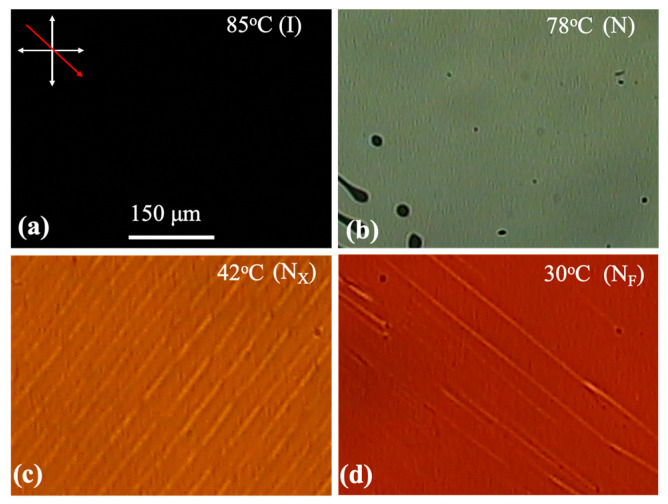
Representative polarizing optical microscopy (POM) textures for a 2 µm cell in the isotropic (I), N, N_X_, and N_F_ phases. (**a**) Isotropic phase, (**b**) nematic phase, (**c**) N_X_ phase, (**d**) N_F_ phase.

Polarizing optical microscopy (POM) studies were carried out using Olympus BX60, Olympus Corp., Tokyo, Japan in sandwich cells between 1 µm and 10 µm thicknesses, with the surfaces treated for planar alignment by PI2555 polyimide coating on the substrates and then rubbing; the substrates were assembled with parallel rubbing directions. This surface treatment and thickness resulted in a uniform planar alignment and made reliable mesophase determination possible. The temperature dependences of the POM images provided the phase sequence (I-79 °C-N-42 °C-N_X_-30 °C-N_F_), which disagrees slightly with the originally reported transition temperatures [33]. The small differences in phase transition temperatures observed are understandable given (a) differences in temperature control performance in various instruments, and (b) that FNLC-919 is a mixture and measurements were performed on different batches as received from the manufacture, which likely do not have exactly matching compositions. Representative textures for a 2 µm cell in each phase are shown in Figure 1.

The texture in the N_X_ phase shows a faint stripe texture perpendicular to the rubbing direction with a periodicity of about 10 μm. POM images where the crossed polarizers are parallel to the rubbing direction and with polarizers uncrossed by ±10° and ±30°, are shown in Figure S1 of the Supporting Information. They show that the director structure in between the defect lines (or walls) is uniformly parallel or perpendicular to the rubbing direction. Uncrossing the polarizers by ±30°, it appears that the defect lines break into oppositely twisted structures. Based on these, we suggest that in adjacent domains the director is slightly tilted (in opposing directions) with respect to the rubbing directions: so-called chevron structures as they have been termed in SmC* materials [37,38]. These have also been reported in the intermediate phase between N and N_F_ in the prototypical material, DIO [6,39], in the antiferroelectric SmZ_A_ phase. We note that in other film thicknesses the stripe system seen in the N_X_ phase of the 2 µm film are much less regular. On cooling the sample to the N_F_ phase, we observe a few lines along the rubbing direction that might indicate domains with opposite polarization along the rubbing direction, as was observed and analyzed before [10,16].

**Figure 2:**
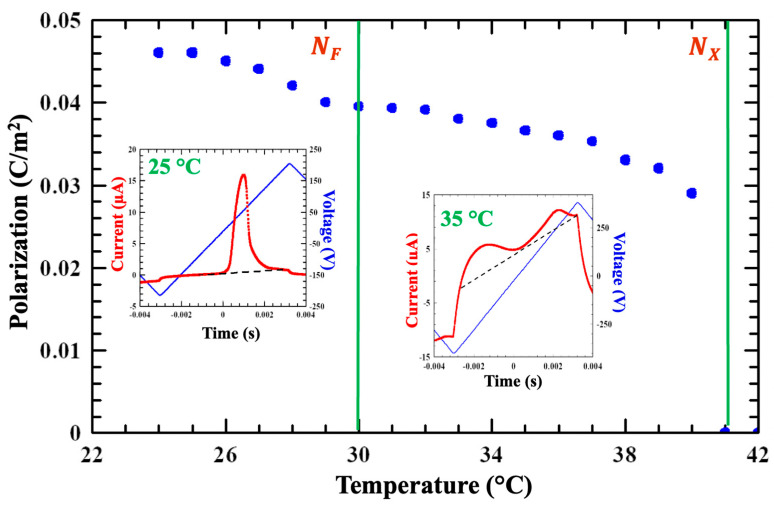
Temperature dependence of the ferroelectric polarization measured on a 10 µm film with in –plane electrodes. Insets show the time dependences of the electric currents at 25 °C in the N_F_ phase and at 35 °C in the N_X_ phase. The vertical green lines indicate where the phase transitions were observed. The black, dashed lines in the insets represent the baseline for establishing the ferroelectricity peaks.

Polarization measurements employed planar cells with 10 µm separation and in-plane electrodes on one substrate separated by 1 mm. This is the standard geometry for accurate measurement of ferroelectric polarization of ferroelectric nematic liquid crystals [10]. The samples were heated to the isotropic phase and then cooled to the N_X_ phase at 1 °C/min. Polarization was determined using the triangle wave technique [40,41] at a frequency of 80 Hz and a maximum potential difference up to 500 V applied between plane electrodes separated by a 1 mm gap. The temperature dependence of the ferroelectric polarization is shown in the main pane of Figure 2. The inset shows representative examples of the ferroelectric and antiferroelectric current peaks.

The polarization is about 0.04 C/m^2^ just below the N-N_X_ transition and increases by about 15% throughout the N_X_ phase. In the N_X_ phase the polarization current shows two distinct peaks: the first where the applied potential difference is negative and the second where it is positive, as shown in the right inset of Figure 2. This signifies antiferroelectricity, where the polarization vector oscillates in space so that the net dipole moment vanishes [37,38]. In the N_F_ phase, the polarization current shows a single peak signifying a true ferroelectric phase (see left inset in Figure 2); the polarization reaches 0.045 C/m^2^ at 25 °C, in agreement with published values [24].

Polarization is directly related to both the molecular dipole and the density. Other materials exhibiting the N_F_ phase have reported densities substantially higher than typical liquid crystals [19,42], even as much as 1.54 g/cm^3^ [38]. Being a mixture, the concept of molecular dipole for FNLC-919 is not straightforward, but we can report its density. Mass density measurements were obtained by placing a pre-weighed mass of FNLC 919 in a precision borosilicate glass capillary with a calibrated bore. The material volume was then obtained by measuring the height of the resulting column. These measurements provided the density of FNLC919 at 25 °C as $\varrho =1.39\pm 0.03\;g/{cm}^{3}$—which is in between previously reported N_F_ materials and the highest ever reported.

**Figure 3:**
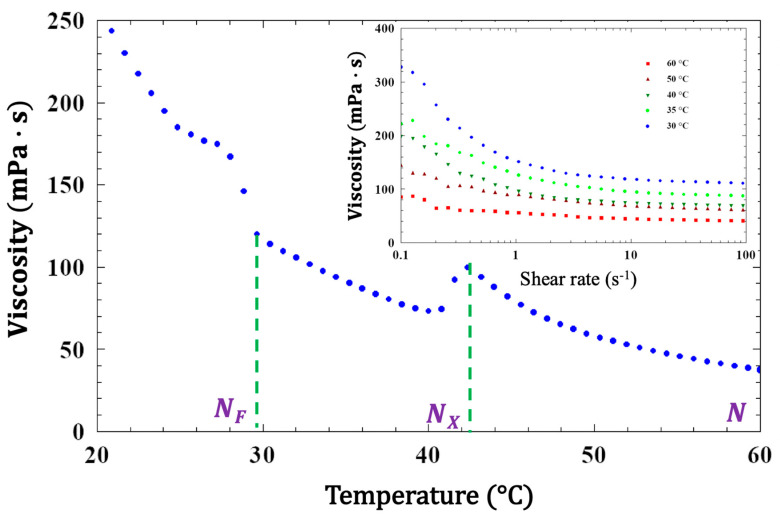
Temperature dependence of the flow viscosity of FNLC 919 at $\dot{\gamma }=700\;s^{-1}$ shear rate. The inset shows the shear rate dependence of the viscosity at various temperatures in the three mesophases in $0.1\;s^{-1}<\dot{\gamma }<100\;s^{-1}$ shear rate range.

Flow viscosity measurements were carried out in a 25 mm diameter cone plate Anton–Paar rheometer with 49 µm cell gap. The temperature dependence of the bulk viscosity is shown in Figure 3 at $\dot{\gamma }=700{\;s}^{-1}$. The viscosity increases on cooling from about 40 mPa·s at 60 °C to 250 mPa·s at 20 °C. Remarkably, the viscosity shows a sharp ~30% decrease upon transition to the N_X_ phase. In the $0.1\;s^{-1}<\dot{\gamma }<100{\;s}^{-1}$ shear rate range, the viscosity decreases at increasing shear rates, indicating shear thinning behavior; this may be evidence of shear alignment. This manner of temperature dependence was also observed in the compound DIO [43].

**Figure 4:**
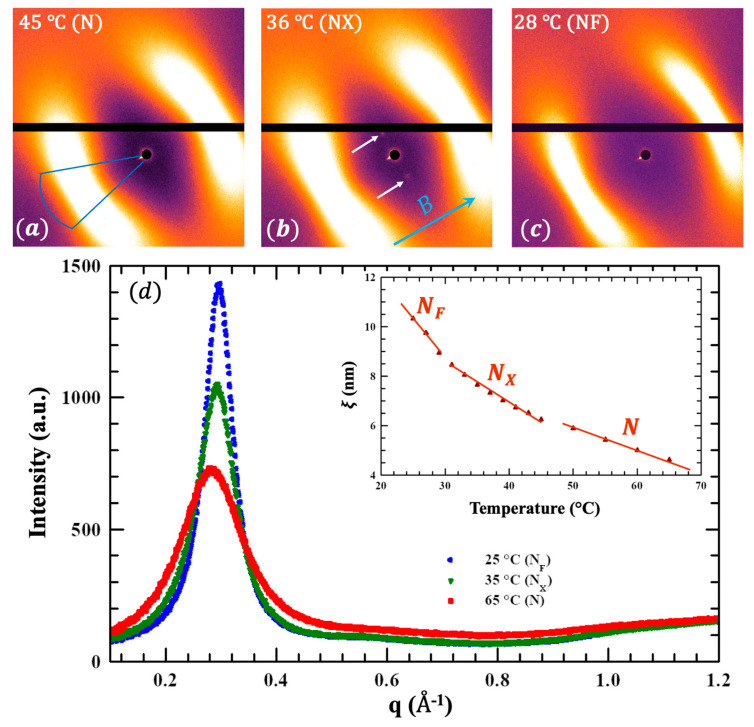
Summary of X-ray measurements. (**a**–**c**) 2D SAXS images (2 h exposure) at representative temperatures in the N (**a**), N_X_ (**b**), and N_F_ (**c**) phases. (**d**) Intensity of diffuse, wider-angle peaks vs. scattering vector q at three temperatures. Inset: Temperature dependence of the correlation length associated with short range positional order of the molecules along the average director. The white arrows in (**b**) indicate weak Bragg spots associated with a mass density wave running perpendicular to the director and consistent with the Sm Z_A_ phase. *B* and the blue arrow depicts the magnetic field direction.

Small-angle X-ray scattering (SAXS) studies were carried out by a Xeuss 3.0 (U)SAXS-WAXS instrument, Xenocs SAS, Grenoble, France. FNLC-919 was filled in a 2 mm X-ray capillary held in a temperature-controlled housing. Samarium cobalt permanent magnets on either side of the capillary (perpendicular to the X-ray beam) provided a constant ~0.5 T magnetic induction. Examples of 2D SAXS images are shown in Figure 4a–c at 45 °C (N phase), 36 °C (N_X_ phase), and 28 °C (N_F_ phase). The blue arrow indicates the magnetic field direction. In all cases we observe diffuse peaks centered at $q\approx 0.3\;{Å}^{-1}$ along the field direction. They indicate spatial correlations on a length scale of $l\approx \frac{2\pi }{3\;{nm}^{-1}\;}\approx 2.1\;nm$.

Figure 4d shows the representative SAXS intensity vs. scattering vector in all three mesophases. For this analysis the intensity was integrated over the area of the blue box in Figure 4a. The spatial correlation length ξ is estimated as 2π over the peak full width at half maximum. ξ vs. temperature is shown in Figure 4d. The lines through the data points show three distinct trends. This shows that there is a subtle although measurably distinct temperature dependence in each phase.

A particularly important finding is shown in Figure 4b, which corresponds to the N_X_ phase. Two distinct (although faint) Bragg peaks are centered at q=0.94 nm^−1^ along the axis, normal to the magnetic field (i.e., normal to the director); this indicates smectic Z-type layering. The *q* value corresponds to l≈6.7 nm. This is precisely the structure found in DIO [43] and several other materials [37] where the SmZ_A_ phase (intermediate between para- and ferroelectric nematic phases) has been confirmed by extensive experimental evidence [37]. These structural studies, along with the demonstration of antiferroelectricity shown in Figure 2 give definitive evidence that the intermediate mesophase in FNLC-919 is positively identified as smectic Z_A_.

**Figure 5:**
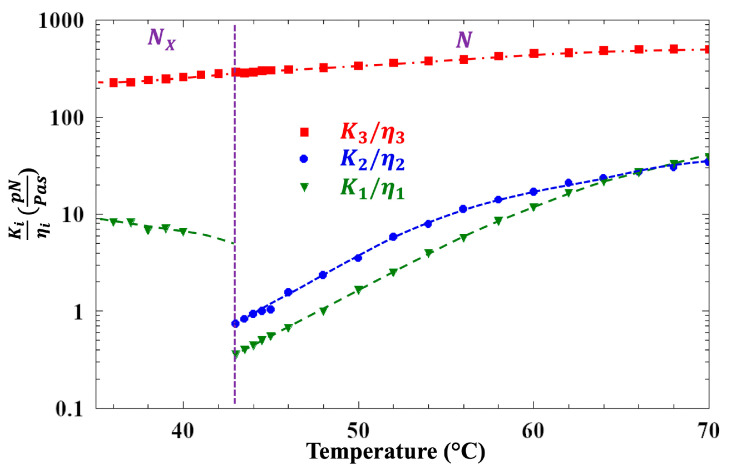
Ratio of relaxation rates to scattering wavenumber squared (Γ/*q*^2^) vs. temperature obtained from light scattering measurements on a 4.6 µm planar-aligned FNLC 919 sample. In the nematic phase, Γ/*q*^2^ corresponds to the ratio of the splay, twist, or bend elastic constant to the corresponding orientational viscosity—namely, $\frac{K_{1}}{\eta_{splay}}$ (green points in N phase), $\frac{K_{2}}{\eta_{twist}}$ (blue points in N phase), and $\frac{K_{3}}{\eta_{bend}}$ (red points in N phase).

Dynamic light scattering (DLS) measurements were conducted on a 4.6 μm thick sample of FNLC-919 contained in an optical sandwich cell treated with parallel rubbed polyimide alignment layers. This geometry is required to (a) minimize static scattering, and (b) eliminate twist domains in the N_F_ phase. The sample was placed in a temperature-controlled oven with optical access and illuminated with 532 nm laser light (focused waist ~30 μm). Time correlation functions of the depolarized scattered light intensity were recorded as a function of temperature for scattering geometries that isolate contributions from the splay, twist, and bend director fluctuations in the nematic phase. The scattered field correlation function in each case was analyzed to extract the relaxation rate (Γ) of the corresponding director fluctuations. The temperature dependence of the ratios of Γ to the square of the scattering vector (*q*^2^) is presented in Figure 5. In the nematic phase, the ratios represent the ratio of the splay, twist, or bend elastic constant to the corresponding orientational viscosity—i.e., $\frac{K_{1}}{\eta_{splay}}$, $\frac{K_{2}}{\eta_{twist}}$, or $\frac{K_{3}}{\eta_{bend}}$. Both $\frac{K_{1}}{\eta_{splay}}$ and $\frac{K_{2}}{\eta_{twist}}$ show strong pre-transitional decreases above the N to N_X_ transition, while $\frac{K_{3}}{\eta_{bend}}$ varies smoothly across the transition. Similarly, the pre-transitional behavior of these parameters was observed in the compound DIO [44]. The absence of any pre-transitional effect on $\frac{K_{3}}{\eta_{bend}}$ is consistent with an N-SmZ_A_ transition, since in this case the layers form parallel to the director, and bend fluctuations would not disrupt the layer structure. In the N_X_ phase, the ratio Γ/*q*^2^, measured in the geometry that corresponds to splay fluctuations in the N phase, increases 20-fold from its value at the transition. As argued in Ref. [44], the splay and twist director fluctuations in the SmZ_A_ phase are coupled due to the unusual layer structure, with the director lying parallel to the layers, and the dispersion of Γ measured in DIO for scattering angles ranging between the normal twist and splay scattering geometries was explained by this coupling. Unfortunately, we were unable to measure Γ reliably in the twist geometry in the N_X_ phase of FNLC919 due to contamination from strong static scattering at a low angle.

**Figure 6:**
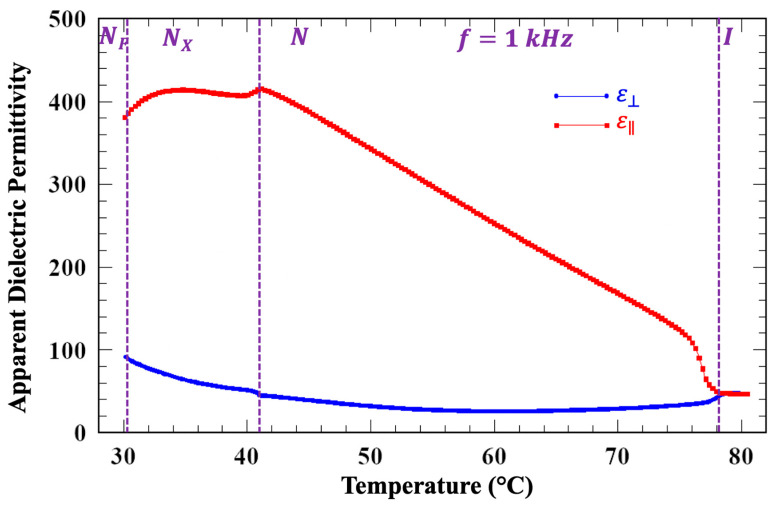
Apparent perpendicular and parallel component of the real part of the dielectric permittivity vs. temperature measured in cooling at 1 kHz under 4 mV input voltage.

To measure the apparent dielectric constants, we measured the current produced by a sine-wave potential in an FNLC-919 sample. Specifically, we used a Princeton Applied Research Model 181 Princeton Applied Research, Oak Ridge, Tennessee, current-to-voltage preamplifier and a Stanford Research Systems 830 lock-in amplifier, Stanford Research Systems, Sunnyvale, CA, USA to simultaneously measure the in-phase and out-of-phase current. For each elastic constant, the cell thickness and rubbing direction were chosen so as to observe the relevant Freedericksz transition (splay, twist or bend) at an attainable external field. The ratio of the out-of-phase current to the voltage then yields the apparent dielectric permittivity. To determine $\varepsilon_{⊥}$ the material was held in a 7.3 μm thick sandwich cell with planar alignment. $\varepsilon_{∥}$ was measured using a cell having 9.8 μm spacing with surfaces treated for homeotropic alignment (in the N and N_X_ phases). For all measurements reported here, the applied potential was 4 mV and 1 kHz. [45]. For both cells, prior to introducing the LC material, the empty cell capacitance was measured. These data do not extend into the N_F_ phase because of the well-documented issues with determining material dielectric constants using capacitance measurements [46,47], as well as the difficulty in obtaining homeotropic alignment in this phase. These results are shown in Figure 6.

In the N phase $\varepsilon_{∥}$ increases from ≈40 at the I-N transition to ≈400, while $\varepsilon_{⊥}$ remains almost constant indicating positive dielectric anisotropy ($∆\varepsilon =\varepsilon_{∥}-\varepsilon_{⊥}>0$) increasing to about 360 at the transition to the N_X_ phase. Such a large dielectric anisotropy is either an artifact related to ferroelectric clusters or due to the large dipole moment (μ), which is likely over 9D.

How might we understand such a large Δε? Since this material does exhibit an underlying ferroelectric phase, the effective molecular dipole μ_mol_ must be fairly large, presumably greater than 9D. Yet, according to Maier-Meier theory [48], this in and of itself is likely not enough. While a large molecular dipole results in large Δε, orientational correlations between dipoles enhance this effect substantially. Roughly, the result for Δε can be explained with a Kirkwood correlation factor (*g*) of about 4. That is, for every antiparallel dipole pair, there are four parallel pairs. This can be contrasted to 5CB, in which *g* is around unity [49]; indeed, water at standard temperature (0 °C) and pressure (1 bar), which is known to be highly coordinated, also has *g* around 4 [50]. It must be stated that the mechanism noted here is presented as being plausible, and certainly not definitive—especially given that we are considering a mixture containing an unspecified number of compounds.

In the N_X_ phase, $\varepsilon_{⊥}$ is increasing and $\varepsilon_{∥}$ is decreasing, and thus the apparent ∆ε is decreasing. The reason for this is less clear, although the measurements in Figure 2 indicate that this phase is antiferroelectric; we can speculate that this may be the effect of decreasing correlation between molecular dipoles. Such an effect would require independent confirmation which is beyond the scope of this work.

**Figure 7:**
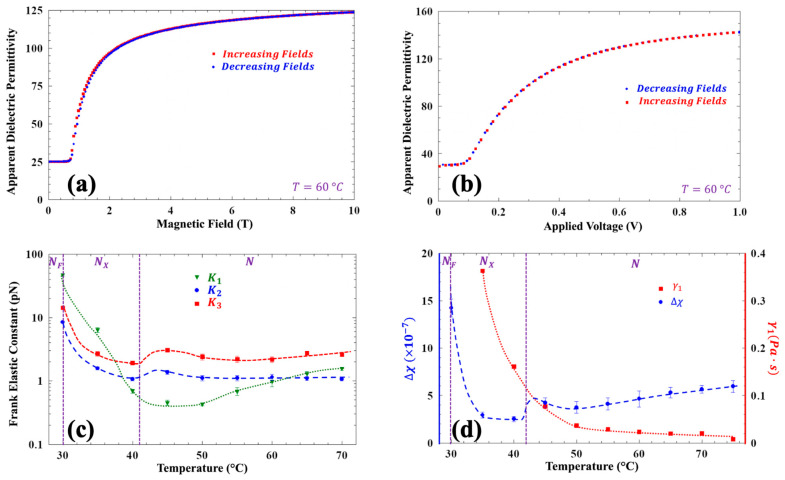
Magnetic field and applied voltage dependences of the apparent dielectric constant, the temperature dependences of the Frank elastic constants, the diamagnetic anisotropy, and the rotational viscosities. (**a**) Magnetic field dependence of the apparent dielectric constant at 60 °C in the N phase; (**b**) 1 kHz sinusoidal applied voltage dependence of the apparent dielectric constant at 60 °C; (**c**) temperature dependences of the Frank elastic constants calculated from Freedericksz measurements in the N and N_X_ phases; (**d**) temperature dependence of the diamagnetic anisotropy calculated from magnetic Freedericksz measurements in the N and N_X_ phases (left axis) and of the rotational viscosity calculated from the relaxation time of the magnetic measurements.

The Frank elastic constants for FNLC-919 were determined via observing the Freedericksz transitions in this material. The results of magnetic and electric Freedericksz transition measurements are shown in Figure 7a and Figure 7b, respectively. The calculated temperature-dependent Frank elastic constants are shown in Figure 7c, and the temperature dependences of the diamagnetic anisotropy and rotational viscosity are represented in Figure 7d. All calculations employed to determine elastic constants are described in Ref [51].

Specifically, in the splay geometry, the threshold voltage *Vth* for the electric field-induced Freedericksz transition is $V_{th}=\pi \sqrt{\frac{K_{11}}{\varepsilon_{0}\;∆\varepsilon }}$; an example is shown in Figure 7b. Using ∆ε from the previously described measurements allows for the determination splay constant, *K*_11_. In the same geometry, when inducing the Freedericksz transition with a magnetic field, the threshold is given by $B_{th}=\frac{\pi }{d}\sqrt{\frac{K_{11\;}\mu_{0}}{\Delta \chi \;}}$, where *d* is the separation between the substrates. Using the value of *K*_11_ from the electric Freedericksz transition measurement, we then obtain diamagnetic anisotropy, ∆χ as shown in Figure 7d.

For the magnetically induced bend and twist Freedericksz transitions, we employed different geometries. For the bend transition, we used homeotropic alignment with a magnetic field parallel to the substrates. For the twist transition, we used planar alignment with a magnetic field parallel to the substrates and perpendicular to the rubbing direction. In both cases, we detected the onset of the Freedericksz transitions via measuring the capacitance. The threshold field for the bend transition is $H_{th}^{B}=\frac{\pi }{d}\sqrt{\frac{K_{33}\mu_{0}}{∆\chi }}$; using the previously determined Δχ we obtained the bend elastic constant, *K_33_.* For the twist Freedericksz threshold measurements, we used a cell with planar alignment (9 µm cell gap) and in-plane electrodes with a separation of 15 µm on one substrate—the rubbing direction was perpendicular to the probe electric field. In the undistorted state, the director is everywhere parallel to the director and so the capacitance will be proportional to ε_||_. Above the twist transition, the director turns away from the probe electric field, and we see a contribution from $\varepsilon_{⊥}$. Even though the precise relationship between the capacitance and twist angle is complex, the onset of twist, i.e., the Freedericksz transition, is plainly observed—c.f. Figure S3. The threshold $H_{th}^{T}=\frac{\pi }{d}\sqrt{\frac{K_{22}\mu_{0}}{∆\chi }}$ gives the twist constant, *K_22_*. All elastic constants in the N and N_X_ phase are shown in Figure 7c. When in the N_F_ phase, as the standard Freedericksz transition is not present, this approach for measuring elastic constants is not applicable.

The relaxation from the Freedericksz transition-induced distorted state provides insight into the orientational viscosity. When the external field is abruptly decreased from a value above the threshold to zero, the effective birefringence decays exponentially, characterized by a time constant τ = $\frac{\gamma_{1\;d^{2}}}{\pi^{2}K_{11}}$, where $\gamma_{1}$ is the orientational viscosity. The representative time dependence of the birefringence upon the electric field removal at 75 °C is shown in Figure S4. Although backflow effects are theoretically anticipated in this configuration, they are not expected to significantly influence the decay time constant [52]. The temperature dependence of the rotational viscosity $\gamma_{1}$ is shown in Figure 7d. It increases from ${\;\gamma }_{1}\sim 0.01\;mPa\cdot s$ at 70 °C in the N phase to ${\;\gamma }_{1}\sim 0.4\;mPa\cdot s$ at 35 °C in the N_X_ phase, without showing a significant change at the N-N_X_ transition. This suggests a continuous change in the molecular ordering across the phase boundary. Furthermore, the absolute values of ${\;\gamma }_{1}$ are comparable to those reported for conventional calamitic nematics.

## 3. Conclusions

The room-temperature N_F_ mixture FNLC-919, made and distributed by Merck Electronics, KGaA, is a prime candidate as a “reference material” for expanded studies of the recently established ferroelectric nematic (N_F_) phase. This material is stable, readily available, and the relevant mesophases lie in convenient temperature ranges, including a room-temperature N_F_ phase. This latter attribute also renders FNLC-919 attractive for the further development of applications exploiting the ferroelectric nematic state, many of which have been proposed.

In this work, we report a comprehensive set of measurements of those material properties, such as temperature-dependent ferroelectric polarization, flow and rotational viscosity, Frank elastic constants, and diamagnetic anisotropy values, that will be necessary to enhance the future use of this material for both fundamental studies and novel technologies.

Additionally, dynamic light scattering measurements show that the N_X_ phase of FNLC 919, which is intermediate between the nematic and ferroelectric nematic phase, match qualitatively with another material (DIO) with a similar, intermediate phase. In contrast to DIO, FNLC-919 exhibits the N_F_ phase at a lower temperature (closer to ambient) N_F_ phase, although with slightly smaller polarization but larger polarization in the SmZ_A_ phase than that in DIO. Small-angle X-ray measurements confirm that the previously labeled N_X_ phase in FNLC-919 indeed has long-range smectic Z positional ordering. Moreover, polarization measurements reveal that the N_X_ phase is antiferroelectric and temperature-dependent viscosity measurements also show similar behavior to the SmZ_A_ of the DIO. We are therefore confident in establishing the previously identified N_X_ phase of FNLC-919 as a useful reference exemplar of the smectic Z_A_ mesophase.

We show that the polarization increases from 3 µC/cm^2^ to 4 µC/cm^2^ in the SmZ_A_ phase and further to 4.6 µC/cm^2^ by reaching the room temperature in the N_F_ phase. The flow viscosity that shows shear thinning behavior increases from 40 mPa·s at 60 °C to 250 mPa·s at a $700\;s^{-1}$ shear rate. Similarly, the rotational viscosity increases on cooling from less than 20 mPa·s at 60 °C to 400 mPa·s in the middle of the SmZ_A_ phase. The twist and bend Frank elastic constant values are similar to those of the conventional N phase, being in the few pN range, whereas the splay constant is below 1 pN in the N phase, then strongly increases in the SmZ_A_ phase to about 50 pN before reaching the N_F_ phase. Unlike in conventional nematic materials where the diamagnetic anisotropy increases on cooling, here we find it to slightly decrease in the N phase, then strongly increase in the SmZ_A_ phase, when approaching the N_F_ phase.

## Data Availability

The original contributions presented in this study are included in the article and Supplementary Materials. Further inquiries can be directed to the corresponding author.

## Supplementary Materials

The following supporting information can be downloaded at: https://www.mdpi.com/article/10.3390/ma18245496/s1, Figure S1: POM images of a 2 µm FNLC 919 film in the N_X_ phase at 36 °C. In the central texture where Θ=0 the crossed polarizers are parallel/perpendicular to the rubbing direction. Top textures at the left (right) correspond polarizers uncrossed by Θ=10° (Θ=−10°). Bottom textures at the left (right) correspond polarizers uncrossed by Θ=30° (Θ=−30°); Figure S2: Phase sequence by polarizing optical microscopy of FNLC-919 ferroelectric nematic liquid crystal for a 1 μm cell. (a) 85 °C in the isotropic phase; (b) 80 °C right below the I-N phase transition; (c) 42 °C in the N_X_ phase; (d) 30 °C in the N_F_ phase; Figure S3: Magnetic field dependence of the capacitance indicative of the twist Freedericksz transition in an in-plane switching cell at 65 °C; Figure S4: Time dependence of the birefringence upon the removal of the electric field at 75 °C. Blue line is a fit corresponding to single exponential function.
