# Supplementary material for: Comprehensive Characterization of a Reference Ferroelectric Nematic Liquid Crystal Material
Source: Materials (Basel). 2025 Dec 6;18(24):5496. doi: 10.3390/ma18245496 (PMC12734485; doi:10.3390/ma18245496)
Supplement: Supplementary file 1 [file materials-18-05496-s001.zip › materials-3988207-supplementary.pdf]

Supporting information

Comprehensive Characterization of a Reference Ferroelectric Nematic Liquid Crystal Material

Ayusha Paul[1], Milon Paul[2], Manisha Badu[1], Arjun Ghimire[1], Netra Prasad[2], Samuel Sprunt[1], Antal Jákli[1,2] and James T. Gleeson[1]

[1]Department of Physics, Kent State University, Kent, OH, 44242, USA
[2]Advanced Materials and Liquid Crystal Institute, Kent State University, Kent, OH, 44242, USA

1. POM images

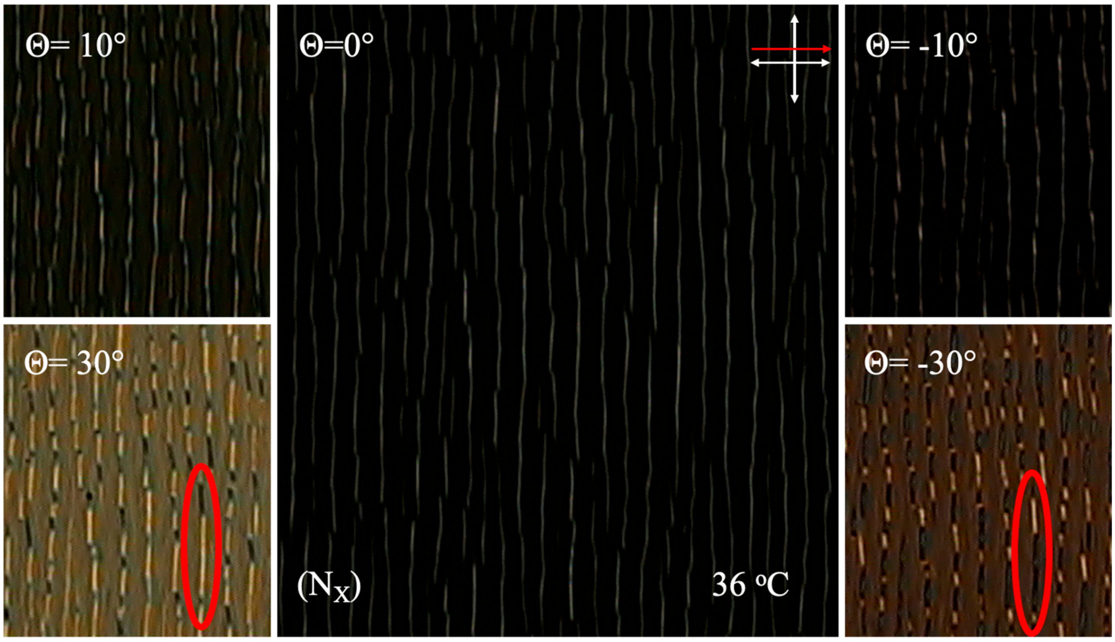

Figure S1: POM images of a 2 µm FNLC 919 film in the NX phase at 36 °C. In the central texture where $\Theta = 0$ the crossed polarizers are parallel/perpendicular to the rubbing direction. Top textures at the left (right) correspond polarizers uncrossed by $\Theta = 10°$ ($\Theta = -10°$). Bottom textures at the left (right) correspond polarizers uncrossed by $\Theta = 30°$ ($\Theta = -30°$).

Figure S1 shows POM images where the crossed polarizers are parallel to the rubbing direction and with polarizers uncrossed by $\pm 10°$ and ± 30 °.

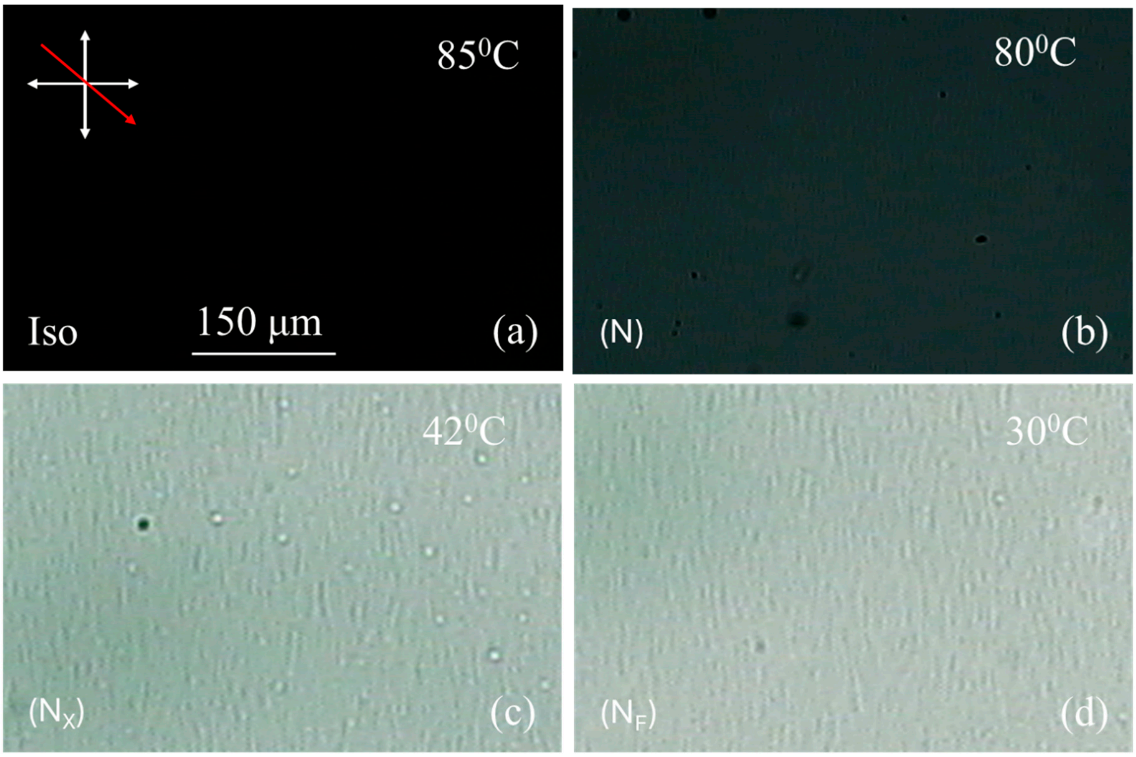

Figure S2: Phase sequence by polarizing optical microscopy of FNLC-919 ferroelectric nematic liquid crystal for a 1µm cell. . (a) 85 °C in the isotropic phase; (b) 80 °C right below the I-N phase transition; (c) 42 °C in the N_X phase; (d) 30 °C in the N_F phase.

Figure S2shows the POM textures at temperatures representative to the different phases of a 1µm cell. In this configuration of cells, we don't see line defects or any domain texture in any of the LC phases. The increasing brightness on cooling is related to the increasing birefringence.

2. Determination of twist elastic constant

The experimental setup was designed to measure the capacitance of FNLC-919 in the presence of a magnetic field. The FNLC-919 sample, prepared on a substrate with interdigitated electrodes, was mounted on a non-magnetic sample holder and placed between the poles of an electromagnet capable of generating magnetic fields up to 1.4 T. The in-plane capacitance of the sample was measured using an LCR meter operating at a fixed 1 kHz frequency with an applied probe voltage of 20 mV. The interdigitated geometry of the electrodes allowed for sensitive detection of capacitance changes in response to the applied magnetic field. To ensure thermal stability during the measurements, the temperature of the sample was controlled using a Lakeshore 331

temperature controller. Shielded BNC cables were used to connect the sample to the LCR meter to minimize noise, especially given the proximity of the sample to the magnetic field source. This setup allowed for precise investigation of the dielectric behavior of the FNLC mixtures under varying magnetic field conditions at controlled temperatures.

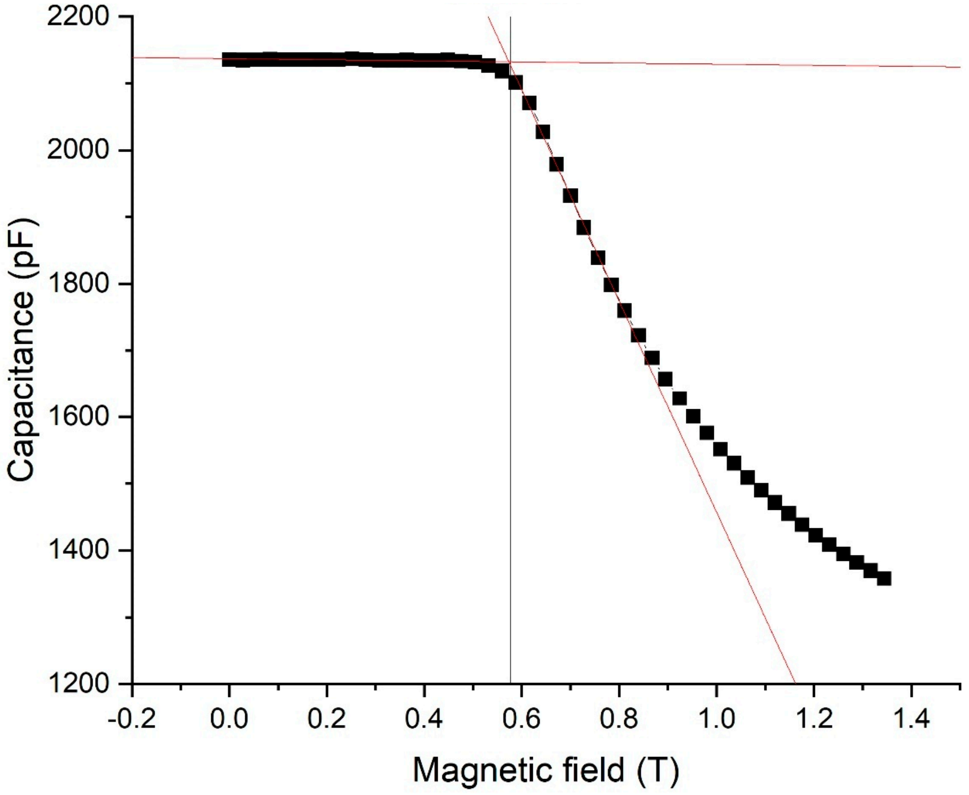

Figure S3: Magnetic field dependence of the capacitance indicative of the twist Freedericksz transition in an in-plane switching cell at 65 °C.

LabVIEW programs were developed to control the experimental setup and directly calculate the dielectric constant of the material. These programs automate the process of applying the input voltage, measuring the resulting current, and computing the dielectric constant based on the capacitance measurements. The lock-in amplifier's ability to precisely measure phase-sensitive signals ensures high accuracy in determining the dielectric properties of the liquid crystal.

The interdigitated electrodes are deposited on one substrate. When a voltage is applied across the fingers, it produces a uniform in-plane electric field between the electrodes. This setup is particularly suitable for studying twist deformations because the in-plane field causes a

reorientation of the liquid crystal molecules along the plane of the substrate, enabling precise measurement of K_{22} through electro-optical or dielectric methods.

3. Determination of rotational viscosity of FNLC-919

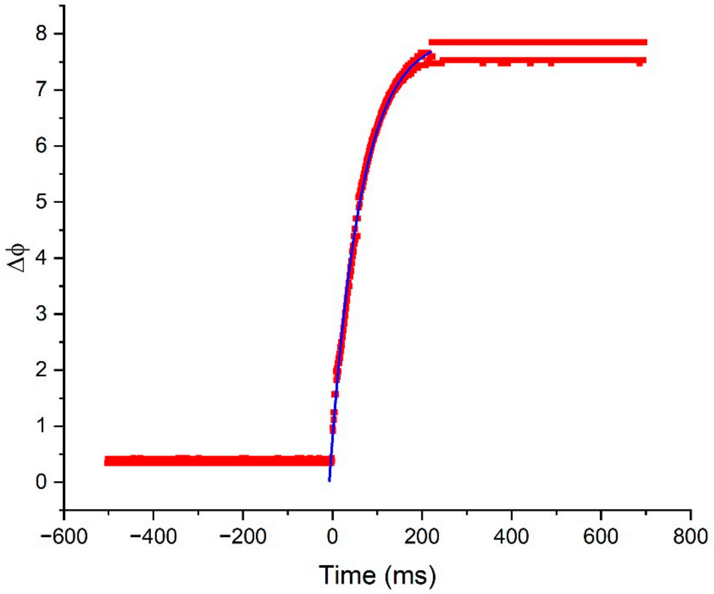

Figure S4: Time dependence of the birefringence upon the removal of the electric field at 75°C.Blue line is a fit corresponding to single exponential function.

Example of how the director relaxation time was determined. At t=0, the ac electric potential was instantaneously zeroed, and the optical phase difference was recorded vs time. The initial increase of phase difference obeys a simple exponential behavior with time constant $\tau = \frac{\gamma_1 d^2}{\pi^2 K_{11}}$. With the earlier measurements of K_{11}, we obtain γ_1
